# Protective and Curative Activities of *Paenibacillus polymyxa* against *Zucchini yellow mosaic virus* Infestation in Squash Plants

**DOI:** 10.3390/biology11081150

**Published:** 2022-07-30

**Authors:** Ahmed Abdelkhalek, Abdulaziz A. Al-Askar, Toufic Elbeaino, Hassan Moawad, Hamada El-Gendi

**Affiliations:** 1 Plant Protection and Biomolecular Diagnosis Department, ALCRI, City of Scientific Research and Technological Applications, New Borg El-Arab City, Alexandria 21934, Egypt; 2 Department of Botany and Microbiology, College of Science, King Saud University, P.O. Box 2455, Riyadh 11451, Saudi Arabia; aalaskara@ksu.edu.sa; 3 Istituto Agronomico Mediterraneo di Bari (CIHEAM-IAMB), Via Ceglie 9, Valenzano, 70010 Bari, Italy; elbeaino@iamb.it; 4 Agriculture Microbiology Department, National Research Centre, Cairo 12622, Egypt; hassanmoawad@yahoo.com; 5 Bioprocess Development Department, Genetic Engineering and Biotechnology Research Institute, City of Scientific Research and Technological Applications, New Borg El-Arab City, Alexandria 21934, Egypt

**Keywords:** *Zucchini yellow mosaic virus*, *Paenibacillus polymyxa*, squash, antiviral, oxidative stress, antioxidative enzymes, gene expression, GC–MS

## Abstract

**Simple Summary:**

*Zucchini yellow mosaic virus* (ZYMV) is one of the most prevalent plant viruses and represents a great challenge for crop production sustainability and human food supplementation. Conventional approaches to disease control depend on the heavy application of hazardous chemicals, which implies severe environmental, animal, and human health challenges. Using natural and microbial products represents a promising tool for sustainable and eco-friendly agricultural applications. The foliar application of the rhizobacteria *Paenibacillus polymyxa* strain SZYM revealed significant enhancements in squash growth parameters and enzyme production when compared to the non-treated plants. On the other hand, there was also a significant decrease in ZYMV accumulation, accompanied with a significant increase in the transcriptional levels of defense-related genes on plants inoculated with *P. polymyxa*. Additionally, a significant decrease in non-enzymatic oxidative stress markers was observed, as well as a considerable increase in reactive oxygen species scavenging enzymes. The present study showed that the *P. polymyxa* strain SZYM could be considered a promising rhizobacterium for enhancing plant growth and defense, and consequently a possible biocontrol agent of plant viral infections.

**Abstract:**

The use of microbial products as natural biocontrol agents to increase a plant’s systemic resistance to viral infections is a promising way to make agriculture more sustainable and less harmful to the environment. The rhizobacterium *Paenibacillus polymyxa* has been shown to have strong biocontrol action against plant diseases, but its antiviral activity has been little investigated. Here, the efficiency of the culture filtrate of the *P. polymyxa* strain SZYM (Acc# ON149452) to protect squash (*Cucurbita pepo* L.) plants against a *Zucchini yellow mosaic virus* (ZYMV, Acc# ON159933) infection was evaluated. Under greenhouse conditions, the foliar application of the culture filtrate of SZYM either in protective or curative treatment conditions enhanced squash growth, reduced disease severity, and decreased ZYMV accumulation levels in the treated plants when compared to the non-treated plants. The protective treatment group exhibited the highest inhibitory effect (80%), with significant increases in their total soluble carbohydrates, total soluble protein content, ascorbic acid content, and free radical scavenging activity. Furthermore, a considerable increase in the activities of reactive oxygen species scavenging enzymes (superoxide dismutase, polyphenol oxidase, and peroxidase) were also found. In addition, the induction of systemic resistance with a significant elevation in the transcriptional levels of polyphenolic pathway genes (*CHS*, *PAL*, and *C3H*) and pathogenesis-related genes (*PR-1* and *PR-3*) was observed. Out of the 14 detected compounds in the GC–MS analysis, propanoic acid, benzenedicarboxylic acid, tetradecanoic acid, and their derivatives, as well as pyrrolo [1,2-a] pyrazine-1,4-dione, hexahydro-3-(2-methylpropyl) were the primary ingredient compounds in the ethyl acetate extract of the SZYM-culture filtrate. Such compounds may act as elicitor molecules that induce systemic resistance against viral infection. Consequently, *P. polymyxa* can be considered a powerful plant growth-promoting bacterium (PGPB) in agricultural applications as well as a source of bioactive compounds for sustainable disease management. As far as we know, this is the first time that *P. polymyxa* has been shown to fight viruses in plants.

## 1. Introduction

Crop losses due to plant diseases are a global threat to human food security and welfare. Food availability and quality should be accounted for among the major scientists’ priorities with the growing world population [1]. The viral infections could develop into severe plant problems with significant crop losses among plant pathogens. The *Zucchini yellow mosaic virus* (ZYMV) is a single-stranded RNA virus in the family *Potyviridae*. It affects most cucurbit crops, such as pumpkin, cucumber, squash, rockmelon, watermelon, and zucchini, causing crop losses of up to 100% when it infects plants before flowering [2,3,4]. Infected plants show a severe leaf mosaic, yellowing blistering, and reduced leaf size, while mottled and twisted areas with uneven coloring are the common infection symptoms in fruits [5,6]. The virus is spread by aphids, which makes it harder to stop the spread of the infection [7]. 

Squash has a very high nutritional content with a lower glycemic index. Though squash fruits are high in vitamins (vitamins B, C, and A), minerals (especially K+), flavonoids, and phenolic compounds, their glycemic index is very low, which encourages their consumption worldwide, particularly in Europe and the Mediterranean regions [8,9]. In addition to the numerous health benefits of squash fruit consumption, several studies have reported the potential antioxidant, anticancer, and antidiabetic applications of squash fruits and flowers [9,10,11]. Considering the high economic importance of squash crops [12,13], extensive efforts have been directed to ZYMV control through cross-protection [14] and developing new resistant squash species [15]. However, the ZYMV remains a serious plant challenge worldwide. Plant viruses find a way to evade and overcome the plants’ resistance mechanisms. Furthermore, heavy agrochemical applications to control pathogens involves important environmental issues.

Plant diseases can be controlled through physical, chemical, and biological approaches. Currently, biological control through plant growth-promoting bacteria (PGPB) represents an economically valuable and eco-friendly solution for controlling and alleviating the consequences of viral infection [16,17]. The PGPB could directly increase the nutrient availability for plant nutrition and improve plant growth and/or stimulate the plants to produce diverse protective phytochemicals against viral challenges [18,19]. Plant infections usually trigger the innate immune system cascade to improve plant resistance. This plant immunity cascade is mediated by two signaling phytochemicals: salicylic acid (SA) and jasmonic acid (JA), according to the infection type and severity. SA regulates the plants systemic acquired resistance (SAR) that includes cell-wall fortification and antioxidant enzyme accumulation in addition to the overexpression of several pathogenesis-related (PR) proteins [20,21,22].

*Paenibacillus* spp. are Gram-positive/negative, rod-shaped, endospore-producing, facultative anaerobic bacteria that were reclassified from Bacillus in 1993 [23,24]. The biocontrol potential of the *Paenibacillus* species has been documented in numerous articles, and recently, *Paenibacillus polymyxa* (*P. polymyxa*) was identified as a biocontrol agent against a wide variety of pathogenic bacteria, nematodes, oomycetes, and fungi [25,26]. However, its antiviral activity is still under investigation. The current study evaluates the protective and curative activities of the foliar application of the *P.* *polymyxa* strain SZYM-culture filtrate (SZYM-CF) against ZYMV infection in squash plants. SZYM-CF was also studied for its effects on plant growth parameters, total soluble carbohydrates (TSC), total soluble proteins (TSP), ascorbic acid (AsA), diphenyl-1-picrylhydrazyl (DPPH), superoxide dismutase (SOD), polyphenol oxidase (PPO), and peroxidase (POX). In addition, the levels of transcription of three pathogenesis-related genes (*PR-1*, *PR-2*, and *PR-3*) and three polyphenolic genes (Phenylalanine Ammonia-Lyase (*PAL*), Chalcone Synthase (*CHS*), and *p*-coumarate 3-hydroxylase (*C3H*)), as well as the ZYMV accumulation level were evaluated. Furthermore, gas chromatography–mass spectrometry (GC–MS) analysis was used to investigate the bioactive constituents of SZYM-CF.

## 2. Materials and Methods

### 2.1. Virus Isolation, Identification, and Molecular Characterization

In 2021, squash (*Cucurbita pepo* L.) leaves showing ZYMV-like symptoms such as a mosaic, yellowing, and leaf deformation were collected from an open field in Alexandria governorate, Egypt. As previously described, the samples were tested for the presence of a ZYMV infection using a complete double antibody-sandwich enzyme-linked immunosorbent assay (DAS-ELISA) kit (DSMZ, RT-0234, Germany) [27]. Squash leaves of DAS-ELISA-positive samples were mechanically inoculated onto *Chenopodium amaranticolor* (*Ch. Amaranticolor*) leaves to develop chlorotic local lesion symptoms [28]. The developed single local lesion at 6 days post-ZYMV inoculation (dpi) was subsequently employed as a source for the ZYMV. The ZYMV isolate was maintained on the squash plants by mechanical inoculation under an insect-proof greenhouse. According to the manufacturer’s instructions, a Plant Virus RNA Kit PVR050 (Geneaid Biotech Ltd., New Taipei City, Taiwan) was used to extract viral RNA from the squash plants. The cDNA was generated from 1 μg of RNA using the Maxima Reverse Transcriptase kit (Thermo Fisher Scientific, Waltham, MA, USA). PCR reactions were carried out with 2 μL of generated cDNA and ZYMV-CP gene-specific primers (Table 1) as previously described [29]. The PCR products were separated on 1.5% agarose gel electrophoresis, stained with ethidium bromide, analyzed using a gel documentation system, purified using a PCR clean-up column kit, and sequenced using an ABI PRISM model 310 DNA sequencer. NCBI-BLAST (http://blast.ncbi.nlm.nih.gov/Blast.cgi) was used to compare the annotated nucleotide sequences to the sequences of previously reported ZYMV isolates. The sequences were then deposited in the GenBank and given an accession number.

### 2.2. Bacterial Isolation, Characterization, and Molecular Identification

As previously described [22], the bacterial isolates were isolated and purified from the rhizosphere soil of the healthy squash plants growing in the Egyptian governorate of Alexandria. Briefly, after removing 3 cm of the soil surface, five soil samples were obtained from 5 to 15 cm at a root depth. Each sample (10 g) was made up of five 2 g samples taken from five rhizospheres of different squash plants growing in the same place. Each sample (10 g) was then agitated in 100 mL of a 0.9% NaCl solution for 30 min. On duplicate nutrient agar plates, 100 µL of each dilution (1 × 10^−4^, 1 × 10^−5^, and 1 × 10^−6^) was streaked aseptically and was incubated at 30 °C for 24 h. Different purified colonies were cultured separately in nutrient broth media for 48 h at 30 °C with 200 rpm shaking. After centrifugation (10 min, 13,000× *g*), the supernatant was collected and filtered with a 0.45 m syringe filter to produce bacterial culture filtrate (CF). The antiviral activity of the bacterial CF was examined on *Ch. Amaranticolor* plants using the half-leaf method [30]. Based on the percentage of inhibition in relation to the number of local lesions, the isolate with the highest effective antiviral activity was subjected to molecular identification and was chosen for further experiments. First, the bacterial isolate was cultivated in a nutrient broth medium under shaking for 24 h. Then, the total DNA was obtained from the cell pellet using a DNA purification kit (Wizard DNA kit, Promega, WI, USA). The genomic DNA was used as a template for 16S rRNA PCR amplification using 16S rRNA universal primers (Table 1) [31]. The resultant 16S rRNA PCR amplicon was electrophoresed in agarose gel, purified using the QIAquick PCR purification kit (Qiagen, Hilden, Germany) and sequenced. The resultant sequence was aligned using CLUSTALW (1.82) and was compared to the sequences in the GenBank database (http://www.ncbi.nlm.nih.gov). The MEGA 11 program was used to create the phylogenetic tree, which was based on the Bootstrap neighbor-joining tree from the CLUSTALW alignment [32].

### 2.3. Greenhouse Experimental Design

Virus-free seeds of squash (*Cucurbita pepo* L.) plants, cultivar Eskandarani, were provided by the Agriculture Research Center, Egypt. Under greenhouse-controlled conditions of 28 °C/16 °C (day/night) with a relative humidity of 70%, the squash seeds were grown in plastic pots (20 cm in diameter). Each pot was filled with 3 kg of mixed sand and clay (1:1) that had been autoclaved before use. On the 15th day after sowing, the size of the plants was carefully monitored to ensure that they were as uniform as possible. The true upper leaves of each plant were dusted with carborundum (600 mesh) and were mechanically inoculated with the ZYMV, using a sodium phosphate buffer (10 mM, pH 7.2) with 0.1% sodium sulfite [33]. The experiment was divided into four groups; each group comprised five replicates. The first treatment group (the mock treatment group) was a group of squash plants that were foliar sprayed with a sterile nutrient broth and mocked with a viral inoculation buffer. The second treatment group (the infected treatment group) was the plant group mechanically inoculated with the ZYMV with foliar spraying of a sterile nutrient broth. The third treatment group (the protective treatment group) was a ZYMV-inoculated plant group sprayed with bacterial CF 24 h before viral inoculation. The fourth treatment group (the curative treatment group) was the ZYMV-inoculated plant group treated by the foliar spraying of bacterial CF 24 h after viral infection. The foliar spray was applied to the complete plant shoots using a hand-held pressure sprayer until drainage occurred and the CF appeared to be coated on the leaves. All the plant groups were maintained for 3 weeks under greenhouse conditions and were monitored daily for symptom development. The squash plants from each group were harvested at 21 days post-ZYMV inoculation (dpi), rinsed several times with water, and assessed for fresh and dry weights (g). For further analysis, the independent biological replicate of each treatment was a pool of 6 squash leaves collected from the 3 plants (2 leaves/plant) in each pot. For an accurate evaluation, each biological replicate was subjected to three separate technical replications. The ZYMV accumulation level was determined using the DAS-ELISA Complete kit. An ELISA was performed on all treatment squash groups to check for systemic viral movement. The optical densities (Ods) were measured at 405 nm with a thermo microplate reader (Multiskan ascent, USA). Absorbance values higher than twice the level of the reactivity of the healthy controls were considered positive for a ZYMV infection. The viral inhibition rate [34] was calculated using the following formula

$$\mathrm{Viral}\;\mathrm{inhibition}\;\mathrm{rate}\;\left(\%\right)=\frac{C-T}{C}\times 100$$

where “C” represents the mean average A405 value of the infected treatment group and “T” represents the average A405 value of each treatment (protective and curative) plant group.

### 2.4. Evaluation of the Total Soluble Carbohydrates and Proteins 

The total soluble carbohydrate (TSC) was evaluated in squash plant groups through the anthrone method as described by Islam et al. [35]. Firstly, squash leaves were homogenized in 95% ethanol in a solid: liquid ratio of 1: 20. After precipitation (at 8000 *g* for 10 min), 100 µL was added to 1 mL of the anthrone solution (200 mg of anthrone in 100 mL of concentrated H_2_SO_4_) and was incubated in a boiling water bath (100 °C) for 10 min. After cooling for 1 h, the reaction absorbance was measured at 625 nm, where the total soluble carbohydrate (mg/g dry weight) was calculated through a standard glucose curve. Furthermore, the total soluble proteins were evaluated in all groups through the Bradford method using a standard curve of bovine serum albumin [36].

### 2.5. Evaluation of Ascorbic Acid Content

Ascorbic acid (AsA) accumulation in the squash plant groups was evaluated through Na-molybdate according to Oser and Hawk [37]. Fresh squash leaf samples were homogenized in sulfosalicylic acid (a solid: liquid ratio of 1: 5). The homogenate was then centrifuged at 13,000× *g* for 15 min. Clear leaves extract (1 mL) was added to 5 mL of the freshly prepared reaction mixture containing 2% Na-molybdate, 0.15 N H_2_SO_4_, and 1.5 mM Na_2_HPO_4_ (2:2:1 *v*/*v*). After incubation at 60 °C for 40 min, the reaction absorbance was measured at 660 nm, where the AsA contents (mg/g FM) were deducted from a standard curve of AsA.

### 2.6. Free Radical Scavenging Activity Evaluation

The free radical scavenging activity was evaluated in different squash plant groups under the ZYMV challenge according to Shimada et al. [38] as follows: 100 µL of plant leaves extract (in a phosphate buffer of pH 7.0) was added to 2 mL of 2,2-Diphenyl-1-picrylhydrazyl (DPPH, 0.05 M in methanol). The color reduction was determined for 30 min at 517 nm and was expressed as the scavenging activity (%) according to the following equation: free radical scavenging (%) = (A_I_ – A_30_/A_I_) × 100, where A_I_ is the initial reaction absorbance and A_30_ is the reaction absorbance after 30 min. 

### 2.7. Antioxidant Enzyme Assays

To elucidate the antioxidant potential of the squash plants under the ZYMV challenge, leave samples were collected from all treatment groups (4 groups) and were assessed for three antioxidant enzyme activities, including superoxide dismutase, polyphenol oxidase, and peroxidase. The leave samples were dried and homogenized in a 0.1 M phosphate buffer (pH 7.0), Na-EDTA (100 mM), and polyvinylpyrrolidone (1% *w*/*v*) with a final solid to liquid ratio of 1:4. The resulting homogenate was then centrifuged (5000 rpm) for 10 min. The clear supernatant was used as a source for different enzymes. First, the superoxide dismutase (SOD) activity was evaluated through a photocatalytic reduction approach using nitro blue tetrazolium (NBT) chloride as described by Beauchamp and Fridovich [39] with some modifications as follows: 100 μL of plant extract was added to a mixture of 75 μM NBT, 13 mM methionine, 0.05 mM EDTA, and 20 μM riboflavin. The final reaction volume was adjusted to 1.5 mL with a potassium phosphate buffer (pH 7.8). Two fluorescent lamps (15-W) were used to initiate the photochemical reaction and the mixture was incubated at 25 °C for 15 min. The reduction in the mixture color was determined at 560 nm, where one unit of SOD activity was defined as a 50% reduction in NBT color. Additionally, the polyphenol oxidase (PPO) activity was evaluated through the quinone method according to CHO and AHN [40] as follows: 0.5 mL of plant extract was added to 1 mL of 50 mM quinone with a pH 6.0 (prepared in 100 mM Tris-HCl) and was incubated for 10 min at 25 °C. At the reaction conditions, the developed color was measured at 420 nm, increasing the absorbance with a 0.001 value representing one unit of the enzyme activity (µM/g of fresh weight). Finally, the peroxidase (POX) activity was determined using guaiacol and hydrogen peroxide as described in Angelini et al. [41]. The assay reaction (1.2 mL) contained: 80 µL of plant extract, 0.5 mL of guaiacol (5 mM), and 120 µL of hydrogen peroxide (1 mM). The final volume was adjusted with a phosphate buffer (100 mM, pH 7.0) and was incubated at 30 °C for 10 min. The developed color was measured at 480 nm and was related to POX activity using a guaiacol extinction coefficient of ε = 26,600 M^−1^ cm^−1^.

### 2.8. The Effect of Foliar CF Application on Squash Plant Gene Expression under ZYMV Challenge

The relative expression levels of three pathogenesis-related genes (*PR-1*, *PR-2*, and *PR-3*) and three polyphenolic genes (*PAL*, *CHS*, and *C3H*) were evaluated through qRT-PCR as previously described [42,43]. Firstly, the total plant RNA was extracted from leaf samples using the RNeasy plant mini kit (QIAGEN, Germany) according to the manufacturer’s instructions and was quantified by the NanoDrop UV spectrophotometer (Labtech International Ltd., Sussex, UK). The purified RNA was used as a template for cDNA synthesis as follows: the DNase I-treated RNA (2 μg) of each sample was reverse transcribed to cDNA using oligo (dT) and random hexamer primers, together with the reverse transcriptase enzyme of Super-Script II (Invitrogen, Waltham, MA, USA), as described previously [22,44]. The cDNA was used as a template for qRT-PCR, using sets of different primers (Table 1), having the *EF1a* gene (housekeeping gene) as a reference to normalize the transcription levels. The qPCR reaction was conducted according to the manufacturer’s instructions of the SYBR Green PCR Master Mix (Fermentas, USA) on the Rotor-Gene 6000 (QIAGEN, ABI System, USA). The relative expression ratio was accurately quantified and calculated according to the ^2−ΔΔ^Ct algorithm [45]. A transcriptional value of > 1 indicates transcriptional up-regulation and values <1 indicate down-regulation.

### 2.9. Identification of CF Bioactive Compounds through Gas Chromatography–Mass Spectrometry (GC–MS) 

The bacterial culture filtrate was subjected to GC–MS analysis to elucidate the bioactive compounds behind the antiviral and growth promotion activities. First, the CF was mixed with ethyl acetate in a ratio of 1:1 and was incubated for 20 min under vigorous orbital shaking (200 rpm). The aqueous layer was retrieved and dried under a reduced vacuum. The dried extract was analyzed through GC–MS (TRACE 1300 Series, Thermo, Waltham, MA, USA), using a mass detector in split mode. Helium gas was used as a carrier (flow rate of 1 mL/min). The injector temperature was 250 °C, while the oven temperature was 60–250 °C for 20 min, with a scanning duration of 0.2 s and a range of 50–650 amu. The CF mass spectra were discovered through comparison to the GC–MS library’s built-in data after 53 min of running time at 70 eV.

### 2.10. Statistical Analysis

The statistical significance of the results was evaluated using GraphPad Prism software using an analysis of variance (ANOVA) at a probability value (*p*-Value) ≤ 0.05. The results represented in the current study are the means (M) of the triplicate experiments with standard deviation (SD) from the means represented as M±SD in tables and error bars in histograms. Statistical significance was indicated with letters in descending order where (a > b > c). Equal statistical significance was indicated with the same letters.

## 3. Results and Discussion

### 3.1. Virus Isolation and Identification

**Figure 1:**
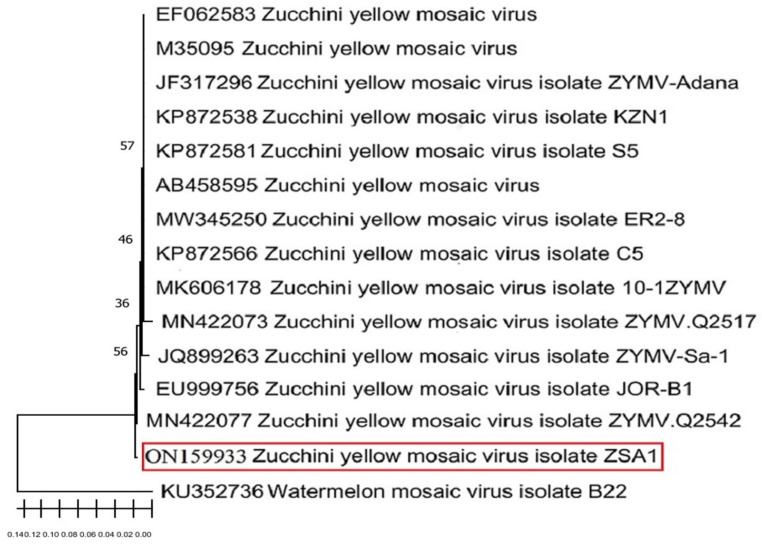
A neighbor-joining phylogenetic tree was constructed based on the nucleotides sequences of *Zucchini yellow mosaic virus* isolate obtained in this study (ZSA1) and those reported in the GenBank.

Almost 94% of the collected squash samples were found to be positive of a ZYMV infection with the DAS-ELISA method. The ZYMV-infected plants exhibited a systemically mild to severe mosaic and squash leaf distortion symptoms, all resembling those of ZYMV infections previously reported [46,47]. A nucleotides sequence analysis of the ZYMV-coat protein (380 bp) showed that our ZYMV isolate (ZSA1) shared 99% of its identity with the other homologues reported in the GenBank, especially with the Australian isolate ZYMV Q2542 (Acc # MN422077); these data were further confirmed by the phylogenetic analysis conducted in this study (Figure 1). The sequence of the ZYMV ZSA1 isolate was deposited in the Genbank under the accession number ON159933.

### 3.2. Bacterial Isolation and Identification 

**Figure 2:**
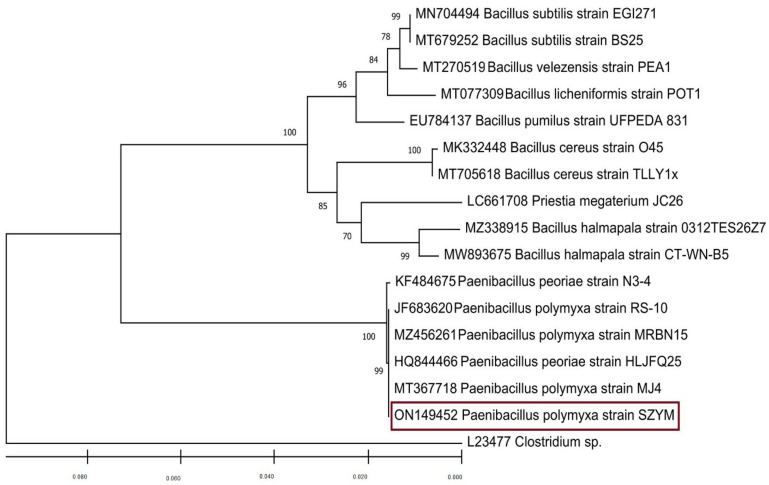
Phylogenic relationship of the locally isolated *Paenibacillus polymyxa* strain SZYM to the other *Bacillus* sp., as represented in the Bootstrap neighbor-joining tree.

Normal rhizosphere microbiota impact plant growth and disease resistance in both direct and indirect ways [48]. Soil from the rhizosphere of squash plants was tested to find biocontrol agents against ZYMV infection. About thirty morphologically diverse colonies were purified and tested for antiviral activity. The bacterial isolate coded SZYM that showed the greatest antiviral activity was selected and subjected to molecular identification. *Paenibacillus* spp. recently emerged as a powerful and promising PGPR that can be widely applied to enhance growth and disease resistance in several important plants [49,50,51]. According to the NCBI-BLAST analysis, the nucleotide sequence of 16S rRNA of SZYM (1468 bp) had a similarity of 99% with other *P. polymyxa* isolates. *P. polymyxa* is commonly found in the rhizosphere of several plants. [51]. Based on the sequence homology results, the bacterial isolate was identified as the *P. polymyxa* strain SZYM, and the annotated sequence was deposited in the GenBank under the accession number ON149452. A phylogenetic tree analysis (Figure 2) showed that SZYM is closely related to other *P. polymyxa* strains, especially to those that were isolated from Pakistan (Acc# MT367718, strain MJ4), China (Acc# HQ844466, strain HLJFQ25), Spain (Acc# MZ456261, strain MRBN15), and Finland (Acc# JF683620, strain RS-10). This means that SZYM is part of the evolutionary lineage of *P. polymyxa* (Figure 2).

### 3.3. Efficacy of SZYM-CF on Symptom Development, Growth Parameters, and Viral Accumulation Level

**Figure 3:**
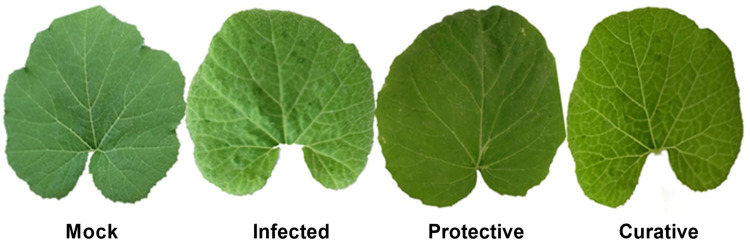
Morphological characteristics of squash leaves under ZYMV challenge in different treatments at 16 days post-ZYMV inoculation. Mock: squash plants foliar sprayed with sterile nutrient broth and mocked with viral inoculation buffer; infected: squash plants mechanically inoculated with ZYMV with foliar spraying of sterile nutrient broth; protective: squash plants sprayed with *Paenibacillus polymyxa* strain SZYM-CF 24 h before ZYMV inoculation; curative: squash plants sprayed with SZYM-CF 24 h after ZYMV inoculation.

Under greenhouse conditions, squash plants mechanically inoculated with the ZYMV exhibited ZYMV-like symptoms at 13 dpi, and severe mosaic symptoms were clearly visible at 15 dpi (Figure 3). Intriguingly, the use of SZYM-CF as a protective or curative treatment caused a delay of roughly five and three days, respectively, in the onset of symptoms. At 16 dpi (Figure 3), mild mosaic symptoms were observed on plants undergoing curative treatment, but no symptoms were shown on plants undergoing protective treatment. On mock-treated squash plants, no symptoms were noticed. In line with how the symptoms showed up, the protective and curative treatments reduced the severity of the disease by 23.15 and 35.27%, respectively. In addition, treatment with SZYM-CF significantly decreased the ZYMV accumulation levels in symptomatic squash leaves. Table 2 shows that the ZYMV accumulation level assessed by DAS-ELISA was the highest at 1.69 for the viral treatment, followed by the protective treatment at 0.34 and the curative treatment at 0.43. The protective activity of SZYM-CF demonstrated encouraging results, with the strongest inhibitory effect (80%). In addition, the inhibition rate for the curative activity was about 75%. Consequently, the results showed that SZYM-CF had different ZYMV infection-inhibiting properties.

The results of the squash growth parameters (Table 2) indicated significant reductions in the fresh and dry weights upon the ZYMV infection (5.78 ± 0.45 and 0.630 ± 0.23, respectively) in the infected treatment group compared to the mock plants (8.09 ± 0.52 and 0.975 ± 0.25, respectively) at 21 dpi. This weight reduction represents about 30% to 35% of both the fresh and dry weights. Plant size reduction and stunting are among the main infection symptoms in the ZYMV that contribute to a total weight reduction [5,52]. Compared to the infected plants, the application of SZYM-CF significantly increased the fresh weights (6.64 ± 0.36 and 6.42 ± 0.39 g) by 14% and 11% in the protective and curative treatment groups, respectively. In the same regard, the squash dry weight was also enhanced in the protective (0.863 ± 0.24) and curative (0.775 ± 0.21) plants to 37% and 23%, respectively, compared to the infected plants (0.975 ± 0.25). Though the plant weight enhancement in the protective and curative treatment groups was significant when compared to the infected treatment group, the values were lower than those of the mock plants. Additionally, the protective treatment increased the plant weights more than the curative treatment, showing how important it is to use SZYM-CF as a preventative measure before an infection for better growth.

### 3.4. Effect of SZYM-CF on Total Soluble Carbohydrates and Total Soluble Protein Content

The evaluation of the total soluble carbohydrates and protein contents in all the treatment groups indicated a significant reduction in these parameters in the infected treatment group to 0.7 ± 0.02 and 1.9 ± 0.01 mg/g DW, which represented about 94% and 24% decreases, respectively, in the total carbohydrate and protein contents, compared to the mock plants. This reduction could be attributed to the leaf size reduction symptoms in ZYMV infections, which directly affect the photosynthesis process and hence the total carbohydrate contents [5]. The foliar application of SZYM-CF enhanced both the carbohydrate and soluble protein contents in the treated groups, with maximum levels in the protective treatment group of about 1.8 ± 0.1 and 2.3 ± 0.4, respectively.

### 3.5. Alternation in Ascorbic acid Content and Free Radical Scavenging Activity

**Figure 4:**
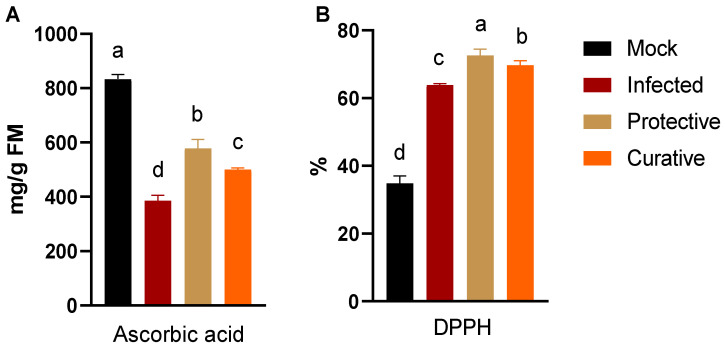
Evaluation of ascorbic acid production titer (**A**) and free radical scavenging activity (**B**) in squash plants. Mock: squash plants foliar sprayed with sterile nutrient broth and mocked with viral inoculation buffer; infected: squash plants mechanically inoculated with ZYMV with foliar spraying of sterile nutrient broth; protective: squash plants sprayed with *P. polymyxa* strain SZYM-CF 24 h before ZYMV inoculation; curative: squash plants sprayed with SZYM-CF 24 h after ZYMV inoculation. The mean values of each column with the same letter do not differ significantly.

Ascorbic acid (AsA) is a major non-enzymatic antioxidant that plays a key role in plant growth and defense [53,54,55]. Though the exact mechanism of AsA in enhancing plants’ resistance to infection is still unclear, the high level of AsA accumulation with the oxidative stress reported in several viral infections strongly asserts its importance [56]. In order to evaluate the potential of SZYM-CF to enhance antioxidant phytochemical accumulation, the AsA level was evaluated in the treated ZYMV-challenged groups compared to the controls, as represented in Figure 4A. The AsA content was greatly reduced upon viral infection to about 55% (386 ± 19 mg/g FM) compared to the mock group (834 ± 16.7 mg/g FM). The foliar application of SZYM-CF alleviated the AsA content in both the protective and curative treatment groups with a significant enhancement in the protective group to 579 ± 32.3 mg/g FM (a 50% increase) compared to the infected treatment group. However, the measured AsA levels in the treated groups were lower than that in the mock plants. In addition to the efficient removal of free radical species, AsA accumulation has been reported to alleviate viral infection symptoms and inhibit RNA virus replication [57,58].

The ability of the squash plants under the ZYMV challenge to scavenge free radicals was evaluated based on the DPPH approach. As shown in Figure 4B, the free radical scavenging activity was significantly enhanced in the infected treatment group to 63.9 ± 0.4%, representing about a 1.8-fold increase compared to the non-challenged control (mock, 34.8 ± 2.2%). The enhancement of the free radical removal potential is a part of the plant’s defense against microbial infection to alleviate the adverse side effects of the surge increase in oxidative stress [59,60]. In the treated groups, the SZYM-CF foliar application enhanced the free radical scavenging by approximately two-fold in both the protective (72.6 ± 1.8%) and curative (69.7 ± 1.3%) groups compared to the mock group. The maximum free radical scavenging activity in the protective plants (72.6 ± 1.8%) represents about a 14% increase compared to the infected treatment group.

### 3.6. Antioxidant Enzymes Evaluation 

**Figure 5:**
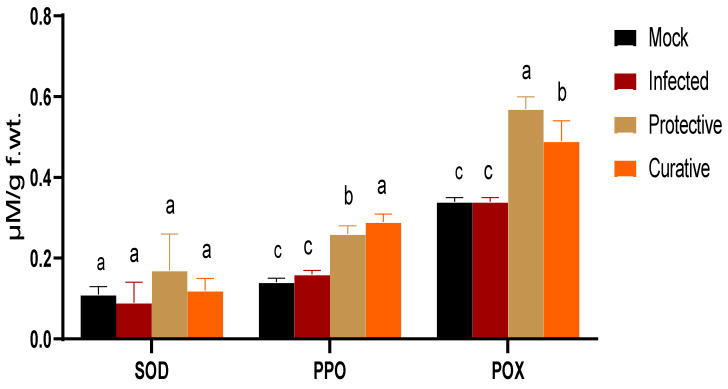
Evaluation of three antioxidant enzymes, including superoxide dismutase (SOD), polyphenol oxidase (PPO), and peroxidase (POX) in squash plants. Mock: squash plants foliar sprayed with sterile nutrient broth and mocked with viral inoculation buffer; infected: squash plants mechanically inoculated with ZYMV with foliar spraying of sterile nutrient broth; protective: squash plants sprayed with *Paenibacillus polymyxa* strain SZYM-CF 24 h before ZYMV inoculation; curative: squash plants sprayed with SZYM-CF 24 h after ZYMV inoculation. The mean values of each column with the same letter do not differ significantly.

High oxidative stress is a hallmark of most plant viral infections; hence, the viral challenged plant accumulates a high level of several antioxidant enzymes to overcome the potential adverse effects of reactive oxygen species accumulation [54,60,61]. In the current study, three antioxidant enzymes were evaluated in squash plants under a ZYMV-challenge as indicators for oxidative stress. As depicted in Figure 5, the SOD level was slightly reduced, with about 22% in the infected treatment group (0.09 ± 0.05 µM/g f.wt.) compared to the mock treatment group (0.11 ± 0.02 µM/g f.wt.). The foliar application of SZYM-CF increased SOD production in both treatment strategies (protective and curative), with a maximum production of 0.17 ± 0.09 µM/g f.wt. in the protective group, representing 55% and 88% increases compared to the mock and infected treatment groups, respectively. The first step in the detoxification of superoxide species (O_2_^−^) is mainly mediated through SOD to generate hydrogen peroxide (H_2_O_2_) molecules that are subsequently hydrolyzed with plant catalases or glutathione peroxidase [22,62].

For PPO activity, the results (Figure 5) indicated a slight enhancement (about 14%) in the PPO level in the infected treatment plants (0.16 ± 0.01 µM/g f.wt.) compared to the non-challenged plants (0.14 ± 0.01 µM/g f.wt.), which could be attributed to the first plant response to viral infection. In the treatment groups, the PPO levels were enhanced by about 60% and 80% in the protective (0.26 ± 0.02 µM/g f.wt.) and curative (0.29 ± 0.02 µM/g f.wt.) groups, respectively, compared to the infected treatment group, which asserts the ability of SZYM-CF to stimulate PPO production in squash plants even after 24 h of a ZYMV infection. In addition to its role in oxygen buffering during the photosynthesis process, PPOʹs defensive role in plant tissue was reported [63,64,65]. PPO mediates the scavenging of many reactive oxygen species by phenolic compounds, generating lignin that is deposited as a physical barrier against pathogens [44,66].

Furthermore, the POX activity was also evaluated in the SZYM-CF treatment groups compared to the controls. POXs comprise a large group of enzymes that participate in many physiological and defensive processes in plant cells. Under plant infection conditions, class III POX plays a major role in lignin formation, the enhancement of cell wall cross-linking, and reactive oxygen metabolism that inhibits pathogen entry and replication [67,68]. The POX titer revealed no change in the infected plants compared to the mock plants, reporting about 0.34 ± 0.01 µM/g f.wt. Upon treatment with SZYM-CF, the POX level was significantly enhanced in both treatment groups: the protective group (0.57 ± 0.03 µM/g f.wt.) and the curative group (0.49 ± 0.05 µM/g f.wt.). The maximum POX production in the protective group represents about a 68% increase in the enzyme level when compared to the control, indicating the efficacy for prophylactic SZYM-CF foliar spraying in POX accumulation. Collectively, the results of the three enzymesʹ evaluation reflect the high potential of SZYM-CF foliar application for alleviating the oxidative stress generated during a ZYMV infection, mainly through the enzymatic pathway, especially with prophylactic application before the viral infection.

### 3.7. Effect of SZYM-CF Foliar Application on Polyphenolic Pathway Synthesis Genesʹ Expression

**Figure 6:**
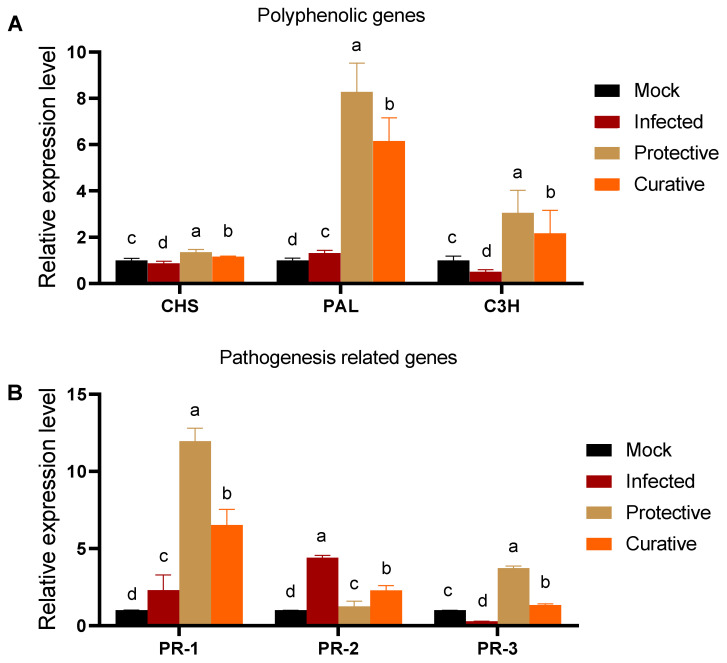
Relative expression levels of three polyphenolics (**A**) and three pathogenesis-related genes (**B**) in squash plants. Mock: squash plants foliar sprayed with sterile nutrient broth and mocked with viral inoculation buffer; infected: squash plants mechanically inoculated with ZYMV with foliar spraying of sterile nutrient broth; protective: squash plants sprayed with *Paenibacillus polymyxa* strain SZYM-CF 24 h before ZYMV inoculation; curative: squash plants sprayed with SZYM-CF 24 h after ZYMV inoculation. The mean values of each column with the same letter do not differ significantly.

The effect of the foliar application of *SZYM-CF* upon the inoculation of the ZYMV on squash was evaluated through monitoring the expression levels of several polyphenolic/flavonoid synthesis enzymes and pathogenesis-related (PR) proteins. Plant chalcone synthase (*CHS*) is a key enzyme that catalyzes the biosynthesis of several flavonoid phytoalexins that are involved in plant protection as a part of the salicylic acid (SA) defense mechanism [69,70]. As indicated in Figure 6A, *CHS* expression was down regulated by about 13% in the infected treatment plants, with a relative expression level 0.87-fold lower than the control. Upon SZYM-CF spraying, *CHS* expression was enhanced in both treatment groups. The protective treatment increased the transcriptional level by about 55% and 35% compared to the mock and infected treatment groups, respectively. Phenylalanine ammonia-lyase (*PAL*) is a crucial enzyme in salicylic acid biosynthesis that regulates several plant cell defensive mechanisms under abiotic and biotic stresses [71]. The results shown in Figure 6A indicated a slight activation of *PAL* expression in the infected treatment group with about 32% (1.32 ± 0.12) compared to the control, which could be attributed to the initial response of the squash plant against the ZYMV infection. Upon treatment with SZMV-CF, the *PAL* expression level surged in the protective and curative treatment groups with about an 8.3- and 6.1-fold increase over that of the mock treatment (Figure 6A) group. The maximum *PAL* expression level was 6.3-fold higher than reported in the infected treatment group, indicating the high potency of SZYM-CF to activate the SA resistance pathway through *PAL* up-regulation. Additionally, the *p*-coumarate 3-hydroxylase (*C3H*) gene was also evaluated in all the plant groups. In the ZYMV-infected group (the infected treatment group), the *C3H* expression was down regulated by about 50% compared to the non-infected plants (the mock treatment group). Interestingly, the treatment with SZYM-CF significantly enhanced *C3H* expression by about 3- and 2.2-fold in the protective (3.05 ± 0.98) and curative (2.17 ± 0.99) treatment groups, respectively. Recent reports have widely addressed the *C3H* protein for plant growth regulation and resistance under many stresses [72,73].

### 3.8. Effect of Foliar Application of SZYM-CF on Pathogenesis-Related Genesʹ Expression 

Under different abiotic and/or biotic stresses, plants usually overexpress several pathogenesis-related (PR) genes that regulate the plants growth and defense to the current challenge [43,74]. Among others, *PR-1*, *PR-2*, and *PR-3* are very important in viral infection, hence they were evaluated in all the treatment groups. As shown in Figure 6B, the ZYMV infection (in the infected treatment group) activated *PR-1* gene expression by 2.3-fold over that of the non-infected plants (the mock treatment group), which could be attributed to the initial response of the squash plants to the ZYMV infection. The results are in accordance with several studies reporting the slight activation of the *PR-1* gene in several plants upon viral infection [43,74,75]. In the SZYM-CF-treated groups, the *PR-1* gene expression was up regulated to 12- and 6.5-fold increases in the protective and curative treatment groups, respectively, compared to the control. The prophylactic application of SZYM-CF before viral infection was superior in *PR-1* overexpression, reporting about a five-fold increase compared to the infected treatment group and reflecting that the SA pathway is the main resistance mechanism in squash plants under the ZYMV challenge. *PR-1* is an SA accumulation marker that regulates systemic acquired resistance (SAR) mechanisms against plant infections [29]. This could be supported by the high titer of *PAL* expression in the SZYM-CF treatment groups as indicated previously (Figure 6A), as *PAL* is a crucial enzyme for SA precursor synthesis [71]. Chen and his colleagues reported a significant reduction in SA accumulation and SA gene markers in *Lotus japonicus* due to *PAL* knockdown [76]. The *PR-1* gene is an essential gene in plant defense to biotic and abiotic stresses, in addition to the regulation of plant growth and development away from stress conditions [21]. 

The *PR-2* gene family includes the *β*-1,3-glucanases that hydrolyze and modify β-glucan, which is a dominant sugar in plant cells and several invading microbes [77]. The released β-glucan units signaled the plant resistance state and enhanced the production of plant phytoalexins as antimicrobial agents [78,79]. Regarding the *PR-2*, the results indicated a significant up-regulation of *PR-2* gene expression in the infected treatment plants, up to a 4.4-fold increase compared to the control. This overexpression could be attributed to the callose-hydrolyzing activity reported for β-1,3-glucanases, which facilitates intracellular viral spreading between adjacent plant cells [80,81,82]. Treatment with SZYM-CF significantly down regulated the *PR-2* gene expression that peaked in the protective treatment group (a 1.26-fold increase) compared to the control. The minimum *PR-2* level in the protective treatment group was about 71% lower than that reported in the infected treatment group, which indicated the ability of prophylactic SZYM-CF spraying to down regulate *PR-2* gene expression and hence the ZYMV internal spread. Furthermore, the expression of the chitinase-encoding gene, *PR-3*, was significantly reduced in the infected treatment plants, up to 71% compared to the mock treatment plants. Treatment with SZYM-CF up regulated the *PR-3* gene expression with 3.7- and 1.4-fold increases compared to the control. *PR-3* encoded chitinase activity and its overexpression is a marker for plant resistance mechanisms mediated through jasmonic acid as a part of induced systemic resistance [20,83]. The *PR-3* role in plant resistance to fungal infection has been widely reported; however, its role in enhancing plant resistance under a viral challenge is still indecisive [84,85]. Zhu and his colleagues reported the necessity of the first induction of JA for SA accumulation in TMV infection, which could explain the reason behind the *PR-3* induction in the ZYMV infection [86]. The results are fully consistent with the increased chitinase activity reported in several plant viral infections [80,87].

### 3.9. Phytochemical Analysis of the SZYM-CF Using GC–MS

**Figure 7:**
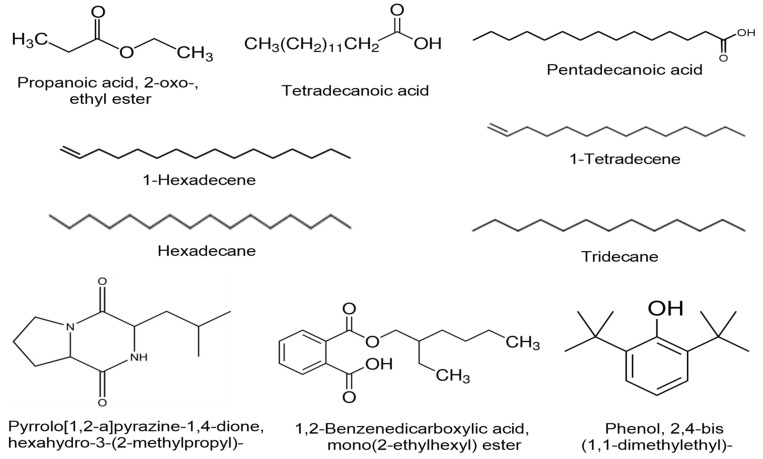
Chemical structures of the ten most abundant bioactive compounds detected in the GC–MS analysis of ethyl acetate extract of culture filtrate of *Paenibacillus polymyxa* isolate SZYM.

The components in the culture filtrate of the SZYM bacterial isolate were subjected to GC–MS analysis to elucidate the compounds behind the antiviral and growth promotion activities. The GC–MS analysis (Table 3) revealed 14 different compounds in the SZYM-CF, including several biologically active aromatic compounds and fatty acids. Figure 7 illustrates the chemical structures of the ten most abundant bioactive compounds identified by GC–MS analysis. Among the detected compounds, two esters, including propanoic acid, 2-oxo-, ethyl ester and benzenedicarboxylic acid, mono (2-ethylhexyl) ester, were detected at a retention time (RT) of 3.43 and 23.40, respectively. The two esters previously revealed diverse biological activities, including antimicrobial, antifungal, antioxidant, and anti-inflammatory potential [88,89]. Benzenedicarboxylic acid, mono(2-ethylhexyl) ester from the marine *Streptomyces* spp. was found to have anticancer activity in vitro against human breast adenocarcinoma (MCF-7) and hepatocellular liver carcinoma (HepG 2) [90]. Tetradecene and Hexadecene are long-chain hydrocarbons that were also detected in SZYM-CF at a RT of 11.03 and 12.74, respectively, with reported antimicrobial activities [91]. Tetradecene revealed several biological activities as reported in the *Lentinus squarrosulus* wild mushroom aqueous extract [92] and also reported from different *Streptomyces* spp. [93]. The GC–MS analysis also revealed the presence of two long-chain alkanes, namely hexadecane and tridecane. Hexadecane (RT of 11.09) was previously reported in the crude extract of *Paracoccus pantotrophus* FMR19, revealing potent antimicrobial activity against *Salmonella* sp., *Proteus* sp., and *Staphylococcus aureus* [94], and also revealed antioxidant activity [91]. Tridecane (RT of 12.79) is one of the main components of *Bupleurum marginatum* oil that revealed significant anti-inflammatory and anticancer effects [95]. Two long-chain fatty acids were also detected in SZYM-CF, including tetradecanoic and pentadecanoic acid at a RT of 14.06 and 15.48. Tetradecanoic acid, also known as myristic acid, is a saturated fatty acid that was recently proved to have antimicrobial activity through targeting ATP-binding cassettes in multidrug resistant *Bacillus subtilis* [96]. Phenol, 2,4-bis(1,1-dimethylethyl)- (RT of 12.18) was reported from *Pseudomonas fluorescens* TL-1 that revealed strong antifungal activity against several phytopathogenic fungi [97] as well as antioxidant activity from *Streptomyces* spp. [98]. Furthermore, Pyrrolo [1,2-a]pyrazine-1,4-dione, hexahydro-3-(2-methylpropyl)- was detected in CF (RT of 14.75) and is a biologically active antioxidant isolated from *Streptomyces mangrovisoli* [99]. The wide range of the biologically active compounds detected in SZYM-CF nominated the *P. polymyxa* isolate SZYM as a powerful PGPR in agricultural applications. It may also explain the reasons behind the potent antiviral activity against the ZYMV reported in the current study; however, further studies are mandatory to specify the exact agent (s) for such antiviral activity. The current study may pave the way for future studies concerning the application of *P. polymyxa* to control other plant viral infections.

## 4. Conclusions

Under greenhouse conditions, the foliar application of the culture filtrate of the *P. polymyxa* strain SZYM isolated from the squash rhizosphere seems to be a promising inducer for systemic resistance in squash against ZYMV infections. When compared to squash plants that were not treated with SZYM-CF, either a preventative or a curative treatment decreased the severity of the disease by up to 23.15% and increased the inhibition index by up to 80%. It also helped the plants grow and increased the amount of their total soluble carbohydrates and total soluble proteins. In addition, the levels of DPPH and antioxidant enzymes (SOD, PPO, and POX) increased in the presence of SZYM-CF in the ZYMV-challenged plants. Furthermore, the induction of systemic resistance with considerable increases in the expression levels of polyphenolic pathway genes (*PAL*, *CHS,* and *C3H*) and pathogenesis-related genes (*PR-1* and *PR-3*) was also reported. Many active compounds such as propanoic acid, benzenedicarboxylic acid, tetradecanoic acid, and pyrrolo [1,2-a] pyrazine-1,4-dione and their derivatives that were detected in the ethyl acetate extract of SZYM-CF could potentially be used as plant growth promoters, defense modulatory agents, as well as for the development of plant-derived compounds to protect plants against viral infections. However, additional studies are needed to confirm these results under field conditions.

## Figures and Tables

**Table 1:** Nucleotide sequences of primers used in this study.

Target Gene	Primer Name	Direction	Nucleotide Sequence (5′ to 3′)
*Zucchini yellow mosaic virus-coat protein*	ZYMV-CP	Forward	GGACAGTGCGACTATAGCTTCAA
		Reverse	TTTAACCGCGAATTGCGTATC
16S ribosomal RNA	16S rRNA	Forward	AGAGTTTGATCCTGGCTCAG GGTTACCTTGTTACGACTT
		Reverse	
Pathogenesis related protein-1	*PR-1*	Forward	CCAAGACTATCTTGCGGTTC
		Reverse	GAACCTAAGCCACGATACCA
Endoglucanase	*PR-2*	Forward	TCAATTATCAAAACTTGTTC
		Reverse	AACCGGTCTCGGATACAAC
Chitinase	*PR-3*	Forward	GGAGGAGTTCTTCAACGGCA
		Reverse	ACGATTGGAGGGCTTCAAGG
Phenylalanine Ammonia-Lyase	*PAL*	Forward	ATGGAGGCAACTTCCAAGGA
		Reverse	CCATGGCAATCTCAGCACCT
Chalcone Synthase	*CHS*	Forward	CACCGTGGAGGAGTATCGTAAGGC
		Reverse	TGATCAACACAGTTGGAAGGCG
*p*-coumarate 3-hydroxylase	*C3H*	Forward	TTGGTGGCTACGACATTCCTAAGG
		Reverse	GGTCTGAACTCCAATGGGTTATTCC
Elongation factor 1-alpha	*EF1a*	Forward	ATTCGAGAAGGAAGCTGCTG
		Reverse	TTGGTGGTCTAAACTTCCAC

**Table 2:** The DAS-ELISA values, growth parameters, and total soluble carbohydrates and proteins of squash plants under ZYMV challenge upon treatment with *Paenibacillus polymyxa* strain SZYM-CF. Mock: squash plants foliar sprayed with sterile nutrient broth and mocked with viral inoculation buffer; infected: squash plants mechanically inoculated with ZYMV with foliar spraying of sterile nutrient broth; protective: squash plants sprayed with *P. polymyxa* strain SZYM-CF 24 h before ZYMV inoculation; curative: squash plants sprayed with SZYM-CF 24 h after ZYMV inoculation.

Treatment	DAS-ELISA Values *	Fresh Weight (g)	Dry Weight (g)	Total Soluble Carbohydrates mg/g DW	Total Soluble Proteins mg/g DW
Mock	0.09 ± 0.02 d	8.09 ± 0.52 a	0.975 ± 0.25 a	11.2 ± 0.6 a	2.5 ± 0.2 a
Infected	1.69 ± 0.06 a	5.78 ± 0.45 d	0.630 ± 0.23 d	0.7 ± 0.02 c	1.9 ± 0.01 b
Protective	0.34 ± 0.03 c	6.64 ± 0.36 b	0.863 ± 0.24 b	1.8 ± 0.1 b	2.3 ± 0.4 a
Curative	0.43 ± 0.03 b	6.42 ± 0.39 c	0.775 ± 0.21 c	1.5 ± 0.1 d	2.1 ± 0.1 ab

* Optical density at 405 nm by DAS-ELISA. The mean values of each column with the same letter do not differ significantly.

**Table 3:** The chemical properties of the 14 compounds of ethyl acetate extract of *Paenibacillus polymyxa* isolate SZYM culture filtrate using GC–MS analysis.

Peak No	Retention Time (RT)	Compound Name	Area	Molecular Formula	Molecular Weight
1	3.43	Propanoic acid, 2-oxo-, ethyl ester (ethyl pyruvate)	1.143.99	C_5_H_8_O_3_	116
2	11.03	1-Tetradecene	253.40	C_14_H_28_	196
3	11.09	Hexadecane	460.90	C_16_H_34_	226.44
4	11.96	Nonane, 1-iodo-	245.18	C_9_H_19_I	254
5	12.18	Phenol, 2,4-bis(1,1-dimethylethyl)-	1.478.91	C_14_H_22_O	206
6	12.74	1-Hexadecene	154.66	C_16_H_32_	224.42
7	12.79	Tridecane	318.84	CH_3_(CH_2_)_11_CH_3_	184.37
8	13.69	Nonane, 5-(2-methylpropyl)-	205.97	C_13_H_28_	184
9	14.06	Tetradecanoic acid	803.98	CH_3_(CH_2_)_12_COOH	228.37
10	14.75	Pyrrolo [1,2-a]pyrazine-1,4-dione, hexahydro-3-(2-methylpropyl)-	214.59	C_11_H_18_N_2_O_2_	210.27
11	15.43	1-Butanamine, N-(1-propylbutylidene)-	610.87	C11H23N	169.31
12	15.48	Pentadecanoic acid	318.68	C_15_H_30_O_2_	242.40
13	15.54	L-Proline, N-valeryl-, heptadecyl ester	994.52	C_27_H_51_NO_3_	437.7
14	23.40	1,2-Benzenedicarboxylic acid, mono(2-ethylhexyl) ester	31.739.38	C_16_H_22_O_4_	278.34

## Data Availability

Not applicable.
